# A comparison of traditional anti-inflammation and anti-infection medicinal plants with current evidence from biomedical research: Results from a regional study

**DOI:** 10.4103/0974-8490.72326

**Published:** 2010

**Authors:** A. Vieira

**Affiliations:** *Applied Sciences, Nutrition Research Laboratory, Kin-9600, Simon Fraser University, Burnaby, BC, V5A 1S6 Canada*

**Keywords:** Ethnobotany, infection, inflammation, medicinal plants, phytochemicals

## Abstract

**Background:**

In relation to pharmacognosy, an objective of many ethnobotanical studies is to identify plant species to be further investigated, for example, tested in disease models related to the ethnomedicinal application. To further warrant such testing, research evidence for medicinal applications of these plants (or of their major phytochemical constituents and metabolic derivatives) is typically analyzed in biomedical databases.

**Methods:**

As a model of this process, the current report presents novel information regarding traditional anti-inflammation and anti-infection medicinal plant use. This information was obtained from an interview-based ethnobotanical study; and was compared with current biomedical evidence using the Medline^®^ database.

**Results:**

Of the 8 anti-infection plant species identified in the ethnobotanical study, 7 have related activities reported in the database; and of the 6 anti-inflammation plants, 4 have related activities in the database.

**Conclusion:**

Based on novel and complimentary results from the ethnobotanical and biomedical database analyses, it is suggested that some of these plants warrant additional investigation of potential anti-inflammatory or anti-infection activities in related disease models, and also additional studies in other population groups.

## INTRODUCTION

Ethnobotanical studies of medicinal plants often provide a first step in the process of selecting plants (or specific phytochemicals) to be tested in experimental models of various diseases. To further warrant such testing, another typical step is to search biomedical databases for existing research evidence that links these plants (or major phytochemical constituents) to the same therapeutic applications. As a model of this process, the current report presents novel information regarding traditional anti-inflammation and anti-infection medicinal plant use in a region of the Iberian Peninsula; and this information is compared with current biomedical evidence.

Over the past 2 decades, there have been several ethnobotanical studies of medicinal plants in the Iberian Peninsula.[1–3] There are no reported, detailed ethnobotanical studies of medicinal plants and their traditional use in the current region of study, the Serras de Aire e Candeeiros (SAC) region of west-central Portugal. Most of this region comprises a national 39,000 ha environmental conservation area, Parque Natural (PNSAC; http://portal.icnb.pt/ICNPortal/). In terms of flora, 2 main publications[4,5] that cover the PNSAC area are known to the author, but these are not primarily concerned with specific, traditional medicinal applications of the plants by the local inhabitants.

The current study provides some novel information regarding traditional medicinal plant use in this region (a more comprehensive ethnobotanical study of medicinal, dietary, and other plant use in the region is underway). Information from plant-application associations in this region is compared for the specific medicinal plants with that in the Medline^®^ biomedical database. Common applications of a given medicinal plant or its main phytochemical constituent(s) are reported and suggested as warranting further analyses in biomedical models or, for comparative purposes, in other regions and populations.

## MATERIAL AND METHODS

Information was obtained from interviews with, and acquired *ad hoc* from, elder residents (*n* = 9, 4 female and 5 male, aged 55–90 years) of villages near the northwest PNSAC border. Plants were identified by several methods: visual inspection, name searches in dictionaries and official botanical websites, and primarily from Flora Digital de Portugal (http://www.jb.utad.pt/pt/herbario/cons_reg.asp), a service provided by the botanical garden of Universidade de Trás-os-Montes e Alto Douro (UTAD) in Portugal.

Analysis of the Medline database (NCBI and National Institutes of Health, USA, Nov. 08; http://www.ncbi.nlm.nih.gov/sites/entrez?db=pubmed) was performed to assess both the extent of overall biomedical research on each of the medicinal plants and biomedical research evidence for the ethnobotanical, plant disease, and association that was identified for each of the plants.

## RESULTS AND DISCUSSION

The compilation of medicinal plants with traditional anti-infection or anti-inflammation uses is shown in Table 1. A total of 10 species are presented, each from a different plant family. To obtain an estimate of the overall level of biomedical research associated with each plant species (or genus, in cases of multiple species), the number of “hits” (publications) for each species based on database searches was obtained and also recorded in Table 1. The most researched plant species include *Allium cepa* and *Sambucus nigra*. Based on the database search, the least researched of the plants identified in this study include *Malva sylvestris, Mentha pulegium, and Capsella bursa-pastoris*.

**Table 1:** Traditional medicinal use of plants for inflammation (InFLA) and infections (InFEC) in the study region

Plant species (Family; local name)	In-FEC	In-FLA	PubMed species/genus	References [InFEC] [InFLA]
*Allium cepa* L.(Alliaceae; cebola)	×	×	796/2115	[8,11,12][11]
*Capsella bursa-pastoris* L.(Cruciferae; ervabom-pastor)		×	57/83	[13]
*Chelidonium majus* L. (Papaveraceae; celidónia)	×		155/193	[10,14,15]
*Ficus carica* L. (Moraceae; figueira)	×	×	78/634	[-][16]
*Malva sylvestris* L. (Malvaceae; malva)	×		19/192	[9]
*Mentha pulegium* L. (Labiatae; poejo)	×	×	25/900	[7] [-]
*Rosa* spp. (Rosaceae; rosa)	×	×	c.100	[9] [-]
*Ruscus aculeatus* L. (Ruscaceae; gil/sbardeira)		×	53/77	[6,17]
*Sambucus nigra* L. (Adoxaceae; sabugueiro)	×		427/549	[18]
*Urtica dioica* L. (Urticaceae; urtiga)	×		225/291	[18]

“References” indicate biomedical research evidence from the database, if any, related to the ethnobotanical usage. ‘PubMed’ indicates approximate number of biomedical research publications on the species/genus (see Methods; if only one number, it refers to genus) present in the database.

References for biomedical research evidence related to the ethnobotanical applications are also indicated in Table 1. The following (a–e) are some of the medicinal plants whose traditional ethnomedicinal applications in this region are corroborated by recently published biomedical research on extracts or phytochemicals of the same plants:

- A steroidal phytochemical of *Ruscus aculeatus*, ruscogenin, has recently been shown to inhibit proinflammatory factors, including tumor necrosis factor-α effects and nuclear factor-κB activation;[6] this plant, butcher’s broom, is used locally for inflammation and arthritis.
- The essential oil of *M. pulegium* (over 70% piperitone/piperitenone) was demonstrated to have antimicrobial activity, especially against gram-positive bacteria, minimum inhibitory concentration (MIC) of approximately 2 µL/mL[7];this plant, pennyroyal mint, is used locally to treat infections.
- There are many publications of antimicrobial activities of *Allium* species, including *A. cepa*, onion, used locally to treat infections. A quercetin derivative isolated from *A. cepa*, 3-(quercetin-8-yl)-2,3-epoxyflavanone, exhibited antimicrobial activity against *Helicobacter pylori* and methicillin-resistant *Staphylococcus aureus*.[8]
- Both *Rosa canina* and M. *sylvestris* were demonstrated to have growth inhibitory activity, IC50 less than 32 mg/mL for biofilm inhibition, against methicillin-resistant *S. aureus*[9]; both plants, rose and mallow, are used locally for infections.
- Inhibitory activity against methicillin-resistant *S. aureus* has also been documented for 8-hydroxydihydro-sanguinarine (MIC of 0.5 mg/mL; and also for other benzo[c] phenanthridine-type alkaloids) extracted from *Chelidonium majus;*[10] this plant, celandine, is used locally to treat topical infections.

In conclusion, the current study provides novel information on medicinal plants with traditional anti-infection and anti-inflammation usage in the PNSAC region. Complementary activities of these plants, or of their phytochemical constituents, are presented. It is suggested that some of these plants, especially *Ruscus* and *Chelidonium*, warrant further investigation of their respective anti-inflammatory and anti-microbial activities in other population groups and in additional experimental models of disease.
